# Three‐dimensional vegetation structure drives patterns of seed dispersal by African hornbills

**DOI:** 10.1111/1365-2656.14202

**Published:** 2024-10-18

**Authors:** Nicholas J. Russo, Docas L. Nshom, António Ferraz, Nicolas Barbier, Martin Wikelski, Michael J. Noonan, Elsa M. Ordway, Sassan Saatchi, Thomas B. Smith

**Affiliations:** ^1^ Department of Ecology and Evolutionary Biology University of California Los Angeles California USA; ^2^ Department of Forestry and Wildlife Technology, College of Technology University of Bamenda Bambili Cameroon; ^3^ Center for Tropical Research, Institute of the Environment and Sustainability University of California Los Angeles California USA; ^4^ Jet Propulsion Laboratory, California Institute of Technology University of California Los Angeles California USA; ^5^ AMAP, Université de Montpellier, IRD, CNRS, INRAE, CIRAD Montpellier France; ^6^ Department of Migration and Immuno‐Ecology Max Planck Institute of Animal Behaviour Radolfzell Germany; ^7^ Department of Biology University of Konstanz Constance Germany; ^8^ Department of Biology The University of British Columbia Okanagan Kelowna British Columbia Canada; ^9^ Okanagan Institute for Biodiversity, Resilience and Ecosystem Services The University of British Columbia Okanagan Kelowna British Columbia Canada; ^10^ Department of Computer Science, Math, Physics and Statistics The University of British Columbia Okanagan Kelowna British Columbia Canada

**Keywords:** hornbill, movement ecology, seed dispersal, step selection functions, tropical forest, UAV‐LiDAR

## Abstract

1. Three‐dimensional (3D) vegetation structure influences animal movements and, consequently, ecosystem functions. Animals disperse the seeds of 60%–90% of trees in tropical rainforests, which are among the most structurally complex ecosystems on Earth.
2. Here, we investigated how 3D rainforest structure influences the movements of large, frugivorous birds and resulting spatial patterns of seed dispersal.
3. We GPS‐tracked white‐thighed (*Bycanistes albotibialis*) and black‐casqued hornbills (*Ceratogymna atrata*) in a study area surveyed by light detection and ranging (LiDAR) in southern Cameroon.
4. We found that both species preferred areas of greater canopy height and white‐thighed hornbill preferred areas of greater vertical complexity. In addition, 33% of the hornbills preferred areas close to canopy gaps, while 16.7% and 27.8% avoided large and small gaps, respectively. White‐thighed hornbills avoided swamp habitats, while black‐casqued increased their preference for swamps during the hottest temperatures. We mapped spatial probabilities of seed dispersal by hornbills, showing that 3D structural attributes shape this ecological process by influencing hornbill behaviour.
5. These results provide evidence of a possible feedback loop between rainforest vegetation structure and seed dispersal by animals. Interactions between seed dispersers and vegetation structure described here are essential for understanding ecosystem functions in tropical rainforests and critical for predicting how rainforests respond to anthropogenic impacts.

## Graphical abstract

This study shows how 3D vegetation structure shapes spatial patterns of seed dispersal by influencing hornbill habitat selection. The seed dispersal modelling framework can be applied to any system with available animal movement data, providing a method to quantify the spatial probability of events occurring due to animal habitat selection.

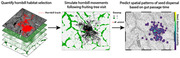

## 1 INTRODUCTION

Seed dispersal by animals is essential for maintaining the structure and composition of tropical rainforests, which harbour over half the planet's carbon stocks and two‐thirds of its biodiversity (Giam, 2017; Pan et al., 2011). Between 60% and 90% of trees in tropical forests depend on animals to disperse their seeds away from specialist predators and competing conspecifics to areas where they can germinate (Janzen, 1970; Rogers et al., 2021; Schupp et al., 2010). Climate and land cover change reduce the structural complexity of rainforests (Saatchi et al., 2021), decreasing their value for wildlife (Deere et al., 2020) and impacting seed dispersal capabilities of trees (Tucker et al., 2021). Although changes in the ecological roles of animals are thought to result from forest degradation, quantifying these relationships has proven challenging (Davies & Asner, 2014; Malhi et al., 2022; Poulsen et al., 2013). A clearer understanding of these relationships can yield insights into possible feedback mechanisms between vegetation structure and seed dispersal by animals (Russo et al., 2023).

Vegetation structure exerts a strong influence on ecosystem functioning by modulating plant productivity (Larue et al., 2019) and influencing animal behaviour (Davies & Asner, 2014). Light Detection and Ranging (LiDAR), a 3D mapping technique, has helped reveal how vegetation structure influences animal behaviour, such as by indicating predation risk (Davies et al., 2016) and reducing energetic costs of movement (McLean et al., 2016; Wittemyer et al., 2019). LiDAR data acquired with unoccupied aerial vehicles (UAV‐LiDAR) enable fine‐scaled characterization of 3D vegetation structure from ground to canopy (Boucher et al., 2023). Attributes of 3D vegetation structure, including height, cover and vertical complexity, are components of ecological niche space that give rise to functional diversity of plants (Valbuena et al., 2020) and habitat partitioning by animals (MacArthur, 1958). Vegetation structure also shapes the distribution of microclimates in ecosystems, reducing temperature extremes and shaping the 3D distributions of animals (Scheffers et al., 2014, 2017). How animals interact with vegetation structure also influences their ecological roles, such as nutrient transport and seed dispersal (Wittemyer et al., 2019).

Animal species differ from each other in habitat selection, but intraspecific variation may underlie additional functional diversity in ecosystems (Shaw, 2020). Population‐level estimates of habitat selection, while useful, may fail to reveal diversity in movements of individual animals (Costa‐Pereira et al., 2022; Shaw, 2020). Individual variation in movement behaviour has important implications for population persistence due to its influence on population connectivity, gene flow, dispersal and colonization events. However, it is less understood how movement variation leads to diversity in ecological roles and resulting effects on ecosystems (Shaw, 2020). Ignoring individual variation in movement behaviour likely weakens the predictive power of models that characterize animal ecological roles (Potts et al., 2022; Potts & Luca, 2023). Consequences of such generalizations can lead to misguided conservation efforts, highlighting the need for intensive sampling of animal populations with high intraspecific variation.

Predictions of wildlife responses to global change are limited by geographical sampling biases. Animal tracking data from the tropics—and particularly Central Africa—are poorly represented in global databases (Kays et al., 2022). However, African hornbill movements have been studied over the past 30 years at increasingly finer spatial and temporal resolution, providing a unique opportunity to study their role in seed dispersal and forest recovery (Chasar et al., 2014; Holbrook et al., 2002; Holbrook & Smith, 2000; Lenz et al., 2011; Mueller et al., 2014). Black‐casqued (*Ceratogymna atrata*) and white‐thighed hornbills (*Bycanistes albotibialis*) are obligate frugivores of mature and degraded rainforests that collectively disperse the seeds of at least 50 tree species (Whitney et al., 1998) and may undertake long‐distance movements of 100 km or more during the food‐lean dry season (Holbrook et al., 2002). These characteristics, combined with gut passage times of up to 6 h, enable hornbills to disperse seeds over long distances (Holbrook et al., 2002; Holbrook & Smith, 2000).

Given that both hornbill species consume fruits of the upper canopy (Hardesty & Parker, 2002) and require large trees for nesting cavities (Stauffer & Smith, 2004), one might expect tall canopies to attract both species. While adult female hornbills are confined to nest cavities for months at a time, they may fly long distances during the longest dry season (December–March) and their annual home ranges average four times greater than adult males (Chasar et al., 2014; Holbrook et al., 2002). Hornbills have a large body size (>1 kg) and must frequently flap their wings to generate lift. Because vegetation structure can increase or decrease energetic costs of movement (Davies et al., 2019; McLean et al., 2016), hornbills may move among vegetation in an energy‐efficient way. For these reasons, we expected 3D vegetation structure to influence hornbill movement behaviour and resulting patterns of seed dispersal.

Here, we explored which attributes of 3D vegetation structure and habitat type influence hornbill movements, examining differences among individuals and between species. We then tested whether vegetation structure influences energetic costs of movement by relating hornbill activity levels to weather conditions and 3D structural metrics of selected habitats. Finally, we simulated spatial patterns of seed dispersal for hornbill‐dispersed trees based on hornbill habitat selection, movement behaviour and gut passage times of seeds, showing how 3D vegetation structure influences seed dispersal by hornbills.

## 2 MATERIALS AND METHODS

### 2.1 Study site

Our study took place in the Dja Faunal Reserve in southern Cameroon, which consists primarily of mature lowland tropical rainforest mixed with inselbergs (rocky outcroppings that rise above the canopy) and *Raphia* palm‐dominated swamps. Spanning 5260 km^2^, it is one of the largest protected areas of Africa's Congo Basin. There are two rainy and two dry seasons annually, with maximum and minimum rainfall occurring in September and May, respectively (Whitney et al., 1998). All hornbills were captured on the 25 km^2^ Bouamir study area located near the centre of the Dja Reserve (3°11′ N, 12°48′ E; maximum elevation 760 m). Bouamir includes a network of former hunting trails and numerous inselbergs that facilitate ground‐based animal tracking (Holbrook & Smith, 2000). A drone‐mounted LiDAR scan of the 25 km^2^ research site was completed in March 2022 using a Dji Zenmuse L1 waveform scanner, with an average point cloud density of 300 points m^−2^.

### 2.2 GPS tracking

Using canopy mist nets (Russo et al., 2024), we captured 16 black‐casqued and five white‐thighed hornbills and tracked each bird with a 27 g (*n* = 18) or 25 g (*n* = 3) solar‐powered transmitter (e‐obs, GmbH, Munich, Germany, www.e‐obs.de) from April 2022 to April 2024. Tag mass was well under 3% of each animal's body mass, the highest recommended percentage (Wilson et al., 2021). Transmitters were attached using a backpack harness made from 1.12 cm (0.44″) tubular Teflon ribbon (Bally Ribbon Mills; Kenward, 2001) with 36.29 kg (80 lb.) strength nylon trammel line (Avinet) reinforcing the interior. The reinforcement was designed to withstand the wear from a hornbill's initial attempt to remove the harness without compromising the bird's well‐being (see Figure S2 for an assessment). All capture and tracking methods were approved by the University of California, Los Angeles Animal Research Committee, under protocol #2019‐037‐01. We performed all field research with permission from Cameroon's Ministry of Scientific Research and Innovation (permit #15/MINRESI/B00/C00/C10/C13) and Ministry of Forestry and Wildlife (permit #1470/PRBS/MINFOF/SETAT/SG/DFAP/SDVEF/SC/ENJ).

All transmitters were programmed to record a GPS fix every 5 min from 5:45 to 18:30 local time, which corresponds with both species' peak period of activity (French & Smith, 2005). At three lower battery levels, the tags were programmed to lower GPS fix rates to 30 or 120 min or 24 h. We retrieved all GPS data using a handheld BaseStation in the study area or remote downloads via local cell networks when birds left the study area. We collected over 250,000 GPS points and 707,000 accelerometer bursts from the tagged hornbills and tracked each hornbill over a period ranging from three to 23 months (Figure S1).

### 2.3 Habitat selection analyses

We conducted an integrated Step Selection Analysis (iSSA) for each hornbill to determine the attributes of vegetation structure that best predict their movements (Avgar et al., 2016; Thurfjell et al., 2014). Variables included canopy height, vertical forest structural complexity, distance to small canopy gaps (≥50 m^2^) and distance to large canopy gaps (≥500 m^2^), all of which were produced at 10 m resolution from the 3D LiDAR point cloud (Table S1). Canopy gaps were defined as areas with no vegetation above 5 m from the ground, consistent with a standard definition of forested habitat (Hansen, 2013). We limited model selection to covariates we expected to influence hornbill behaviour, and covariates with a high Pearson correlation coefficient (>0.6) were not included together in models to avoid collinearity between covariates (Table S2). This practice resulted in the use of a single metric for vertical complexity. Based on field observations and initial data exploration, we hypothesized that black‐casqued hornbills select swamps more often during hotter afternoon temperatures. Accordingly, we included a term for the interaction between temperature and swamp habitat (taking on 0 or 1) in iSSAs for hornbills of both species. Swamp habitats were classified based on a Convolutional Neural Network applied to a cloud‐free Sentinel‐2 image (Brodrick et al., 2019). Temperatures were measured using a DAVIS Vantage Pro 2 weather station (Deblauwe et al., 2023) within the study area every 15–60 min throughout the hornbill tracking period and matched to hornbill GPS data based on timestamp. We note that temperature was recorded in an area of open canopy rather than swamp habitat, and that diurnal temperatures vary among habitat types. We scaled and centred canopy height, vertical complexity index, temperature and both ‘distance to gap’ variables prior to model fitting.

We resampled all hornbill tracks to a 30 min fix rate, then used an iSSA to compare the habitat metrics at the destination of an observed step to those of 10 alternative steps while accounting for movement behaviour (step length and turning angle; Signer et al., 2019). Habitat selection results were insensitive to a higher number of random control steps (*n* = 100 steps; Figures S3 and S4), so we continued analyses with 10 random steps for the sake of computing efficiency. The randomly generated steps represented paths to habitats potentially available to the animal, whose lengths and turning angles are drawn from the observed distributions. Each iSSA contained a habitat selection function that estimated the animal's preference for habitat characteristics:

$$w\left(x\right)=\exp\left(\beta_{1}x_{1}+\beta_{2}x_{2}+\beta_{3}x_{3}+\ldots \beta_{n}x_{n}\right)$$

where $w\left(x\right)$ is a value approximating the likelihood of a location being chosen, $x_{n}$ are the predictors (i.e. habitat metrics) and $\beta_{n}$ are the model coefficients estimated by fitting a conditional logistic regression (Fieberg et al., 2021; Thurfjell et al., 2014). We fit models using the ‘fit_issf’ function in the ‘amt’ R package (version 0.2.1.0; Signer et al., 2019). We only report results from analyses with no issues estimating β coefficients and 95% confidence intervals; as a result, only hornbills with at least 1000 GPS locations within the study area were used in the analysis (*n* = 18).

We estimated population‐level coefficients for black‐casqued and white‐thighed hornbills using generalized linear mixed‐effects models (GLMMs) that avoid pseudoreplication by fitting random slopes for each individual (Muff et al., 2020). We used the same model structure as the individual iSSAs to generate selection coefficients and 95% confidence intervals for each covariate. To further illustrate the interaction between temperature and selection for swamp habitat, we binned temperatures into four categories that correspond roughly to quartiles of the range of temperatures experienced by the hornbills on the study site (<22, 22–25, 25–27 and >27°C) and estimated each species' strength of selection for swamp habitats. All GLMMs were fit using the ‘glmmTMB’ R package (version 1.1.7; Brooks et al., 2023). We also estimated means of each coefficient using inverse‐variance weighted linear modelling (Dickie et al., 2020) and tested for differences between species using ANOVA.

### 2.4 Behavioural valuation of the landscape

We used accelerometer measurements to address the hypothesis that 3D vegetation structure influences energetic costs of flight. Each tag recorded a burst of accelerometer measurements every 10 min from 5:50 to 18:30 local time. Bursts consisted of a raw measurement in the *x*, *y* and *z* axis every second for 20 s. We converted raw accelerometer values to units of gravity (g) and then calculated Overall Dynamic Body Acceleration (ODBA) using the default transformation for e‐obs tags within the ‘ACCstats’ function in the ‘moveACC’ R package (Scharf, 2022). ODBA is the non‐vectorial sum of absolute acceleration values of all three axes, and because it is related to activity levels, ODBA serves as a useful proxy for energy expenditure (Lopez‐Lopez et al., 2022).

We used linear mixed‐effects models to investigate predictors of ODBA, including all 3D structural variables used in habitat selection analyses, as well as rainfall (mm), temperature and a binary variable for swamp habitat. We fitted a model to each hornbill species separately, with individual hornbill ID as a random effect, using the ‘lme4’ R package (version 1.1.34; Bates et al., 2015). For each species, we generated a model with each combination of predictors and ranked all candidate models using the Akaike information criterion corrected for small sample size (AICc), implemented with the ‘dredge’ function in the ‘MuMIn’ R package (version 1.47.5; Bartoń, 2023).

### 2.5 Modelling seed dispersal

**FIGURE 1:**
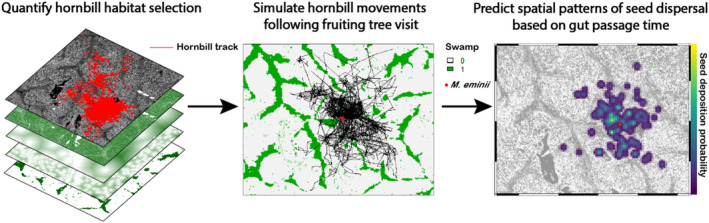
Workflow for generating spatially explicit seed shadow models based on hornbill habitat selection and gut passage times of seeds.

We derived seed dispersal models for six combinations of hornbill and tree species, incorporating gut passage time of seeds and population‐level means of habitat selection. Gut passage times were obtained from a captive feeding experiment involving both species of hornbill (Holbrook & Smith, 2000). For example, *Staudtia kamerunensis* seeds passed through the digestive tract of a black‐casqued hornbill 345 ± 39 min after consumption and 162 ± 8 min for white‐thighed. We developed a simulator that predicts seed dispersal patterns for a fruiting tree based on hornbill movement behaviour, selection for attributes of 3D vegetation structure, and gut passage time of seeds (Figure 1). For each hornbill species, we estimated population‐level selection for attributes of 3D vegetation structure using a GLMM that treats individual hornbill ID as a random effect (Muff et al., 2020), but these models were agnostic to time and therefore did not contain an interaction term for temperature and swamp habitat. Because simulations themselves are used to evaluate the goodness‐of‐fit of step‐selection models (Signer et al., 2024), we evaluated the movement models used for seed dispersal simulations by comparing the distribution of habitat covariates at simulated locations to those of both observed and available locations, thus blending the approaches of (Fieberg et al., 2018) and (Fieberg et al., 2024). This approach is a visual check as to whether each hornbill species selected for or against a habitat feature at the population level, and whether this relationship is captured well by the simulations. We then generated a redistribution kernel from iSSA coefficients that influenced habitat selection significantly (*p* < 0.05) and simulated 100 hornbill trajectories per tree using the ‘amt’ package (version 0.2.0.1; Signer et al., 2019, 2024). Although fruiting trees vary widely in total frugivore visits, many trees receive at least 100 visits during their fruiting period (French & Smith, 2005). We simulated spatial patterns of seed dispersal surrounding the crown of one individual of three different tree species in the hornbill diet (*Staudtia kamerunensis*, *Xylopia hypolampra* and *Maesopsis eminii*) as a Poisson point process with intensity *λ* (Baddeley & Turner, 2005). Tree locations were selected from a network of individuals monitored for a concurrent project on fruiting phenology. Each simulation included 50 movement steps of 20‐min duration, with the starting turn angle based on a random uniform distribution. For each simulation, we assigned a probability of seed deposition at each movement step by fitting a gamma distribution to the gut passage times and standard errors reported by Holbrook and Smith (2000). We estimated and smoothed kernel density estimates of seed dispersal events by weighting each movement step according to the probability of deposition based on gut passage time using the ‘spatstat’ package (version 3.0–6; Baddeley & Turner, 2005). The final product for each hornbill‐tree pair (*n* = 6) was a spatially explicit map of seed dispersal events based on 100 simulated trajectories originating from a fruiting tree. All analyses were conducted using R version 4.3.1 (R Core Team, 2023).

## 3 RESULTS

### 3.1 Hornbill habitat selection

**FIGURE 2:**
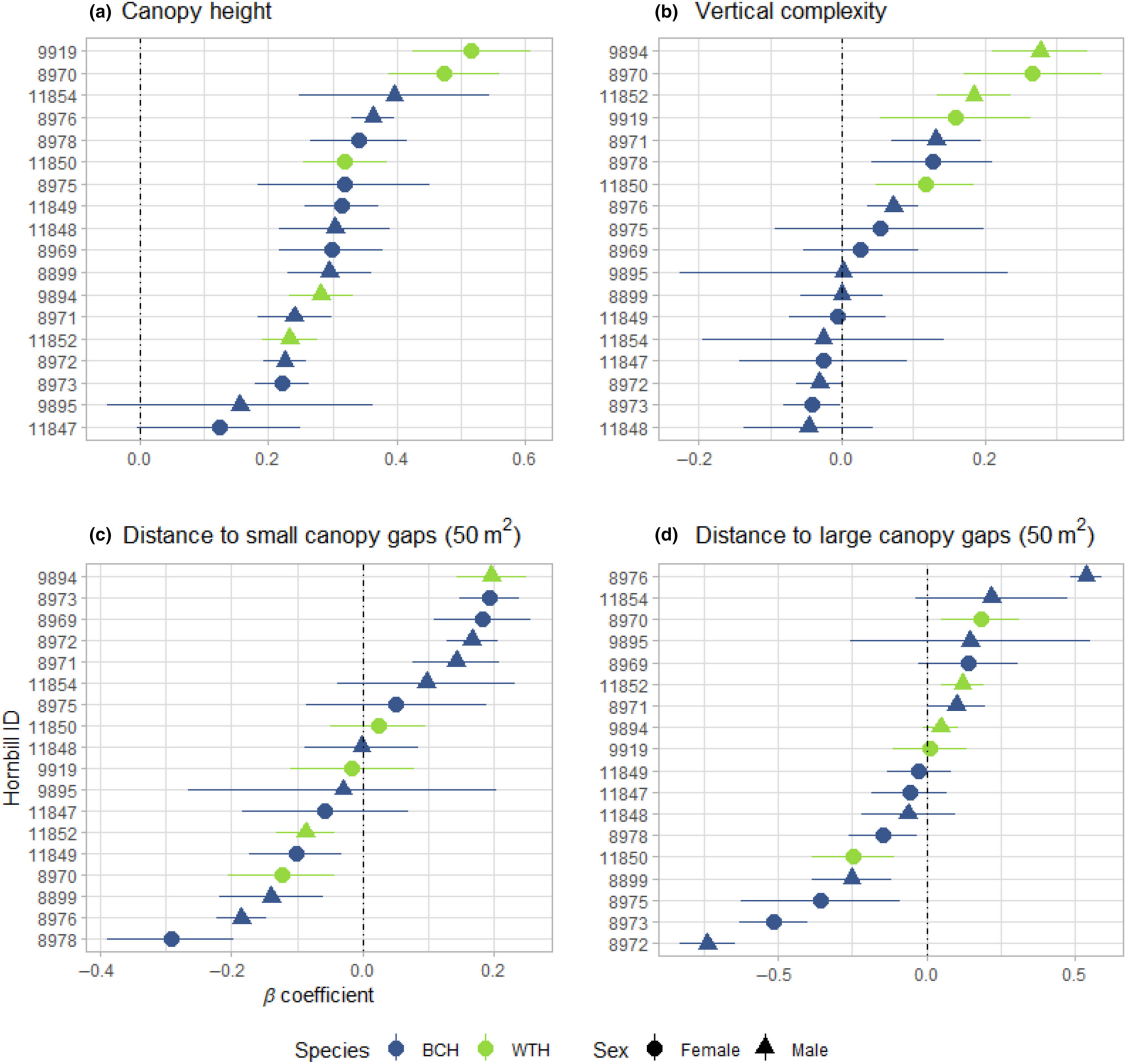
Hornbill selection for 3D structural attributes at 10 m resolution, including (a) canopy height, (b) vertical complexity index, (c) distance to small canopy gaps (≥50 m^2^) and (d) distance to large canopy gaps (≥500 m^2^). Coefficients of habitat selection (*β*) and 95% confidence intervals for each individual hornbill in the study are based on an integrated step selection analysis (BCH, black‐casqued hornbill; WTH, white‐thighed hornbill). The dotted line at *x* = 0 in each plot represents no selection. Note that the range of values in the *x* and *y* axes differs for each plot.

All hornbills preferentially moved among areas with greater canopy height except for one hornbill that frequented the open‐canopy research camp (ID: 11847) and another that was tracked on the study site only as a juvenile (ID: 9895; Figure 2a). Eight hornbills selected habitats with greater vertical complexity (Figure 2b). At the population level, only white‐thighed hornbills preferred habitats with higher vertical complexity (Table 1). Based on coefficients estimated from inverse variance‐weighted linear modelling, we found that white‐thighed hornbills were associated with forests of greater canopy height (ANOVA: *F*_2,16_ = 130.13, *p* < 0.001) and vertical complexity (ANOVA: *F*_2,16_ = 19.6, *p* < 0.001) than black‐casqued.

**TABLE 1:** Population‐level estimates (*β*) of habitat selection and movement behaviour for both hornbill species.

Covariate	BCH	WTH
Canopy height	0.260 (0.018)***	0.337 (0.042)***
Vertical complexity index	0.019 (0.018)	0.189 (0.026)***
Distance to gap ≥50 m^2^	−0.023 (0.035)	−0.0002 (0.047)
Distance to gap ≥500 m^2^	−0.043 (0.050)	−0.009 (0.035)
Swamp	0.379 (0.074)***	−0.708 (0.092)***
Temperature: Swamp	0.160 (0.018)***	0.079 (0.072)
log(Step Length + 1)	0.002 (0.009)	−0.012 (0.010)
cos(Turn Angle)	−0.278 (0.087)***	−0.120 (0.028)***

*Note*: Estimated coefficients of selection and standard error (in parentheses) for each predictor of movement steps are based on a generalized linear mixed‐effects model with random slopes for each individual hornbill. The number of asterisks (*) after a coefficient estimate corresponds to significance at the level of 0.05(*), 0.01(**) and 0.001(***), respectively.

Abbreviations: BCH, black‐casqued hornbill; WTH, white‐thighed hornbill.

**FIGURE 3:**
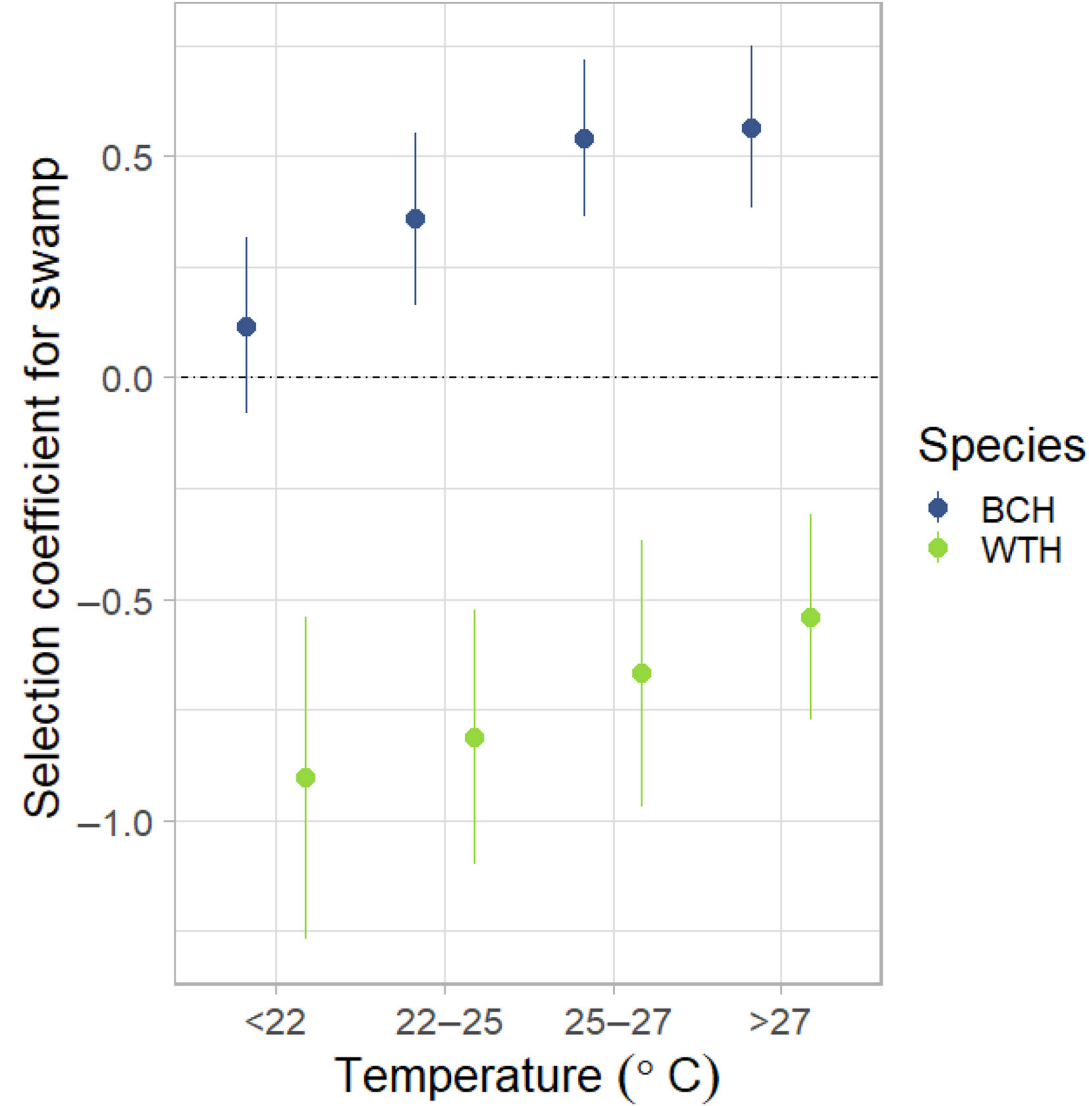
Population‐level selection coefficients for swamp habitat (*β*) with respect to ambient temperature, based on generalized linear mixed‐effects models fit to each of four temperature bins per species. Points and bars represent estimates and 95% confidence intervals, respectively. The dotted line at *y* = 0 represents no selection. BCH, black‐casqued hornbill, WTH, white‐thighed hornbill.

**FIGURE 4:**
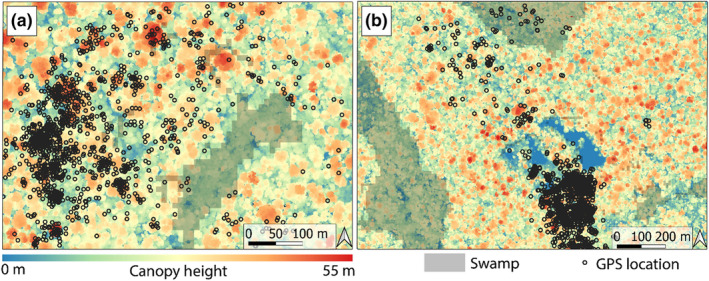
GPS locations of a (a) male white‐thighed hornbill (ID: 9894) and (b) male black‐casqued hornbill (ID: 8972), for June 2023 overlain on a map of canopy height (1 m resolution) with swamp habitats delimited in grey. The maps show both hornbills' preference for the tallest trees of the landscape, the white‐thighed's avoidance of a swamp and the black‐casqued's regular use of a swamp. The extensive area of low canopy height (blue colour) in panel (b) is an inselberg and because the home range of 8972 is associated with the inselberg, this hornbill's iSSA revealed a preference for habitats near large canopy gaps.

Hornbills showed a variety of responses to both large and small canopy gaps. Six hornbills (33.3%) selected areas closer to small canopy gaps, while five (27.8%) appeared to avoid them (Figure 2c). Three of the hornbills (16.7%) appeared to avoid large canopy gaps (Figure 2d), which included inselbergs, the research camp and large treefalls. Six hornbills (33.3%) were attracted to these landscape features. Notably, three birds preferred areas closer to small gaps while avoiding large gaps (ID: 8970, 8976, 11852) and two birds displayed the opposite relationship (ID: 8972, 8973). In addition, we detected no significant response of either species to small or large canopy gaps at the population level (Table 1). Black‐casqued hornbills were more likely to use swamps during hotter temperatures, while white‐thighed hornbills avoided them (Table 1; Figure 3). Black‐casqued hornbills' preference for *Raphia‐*dominated swamp forests and white‐thighed hornbill's tendency to avoid these habitats is a major behavioural difference between the two species (Figure 4).

### 3.2 Behavioural valuation of the landscape

Black‐casqued hornbills exhibit their lowest activity levels—as measured by ODBA—during hotter temperatures (Figure S5A) and when they were within swamp habitats (Figure S6). White‐thighed hornbills also exhibited their lowest activity levels during hotter temperatures (Figure S5B) but no other predictors were included in the top GLMM. We found clear support for the top model of both black casqued (Table S3) and white‐thighed hornbill ODBA (Table S4), with the second‐best model differing by 8.42 and 6.15 AIC_c_ units, respectively. No 3D structural variables were consistently included in top models of ODBA for either species.

### 3.3 Modelling seed dispersal

**FIGURE 5:**
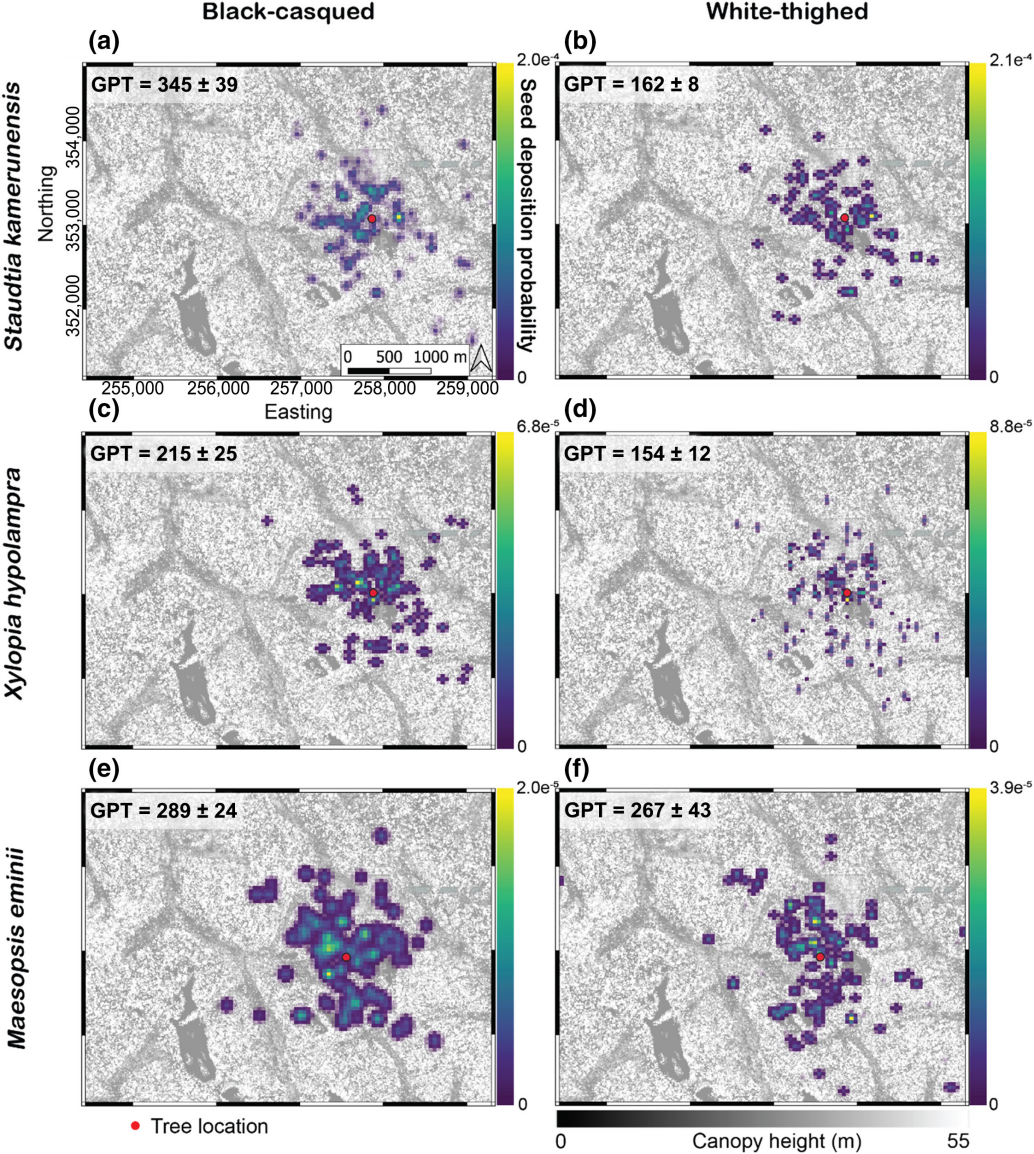
Comparing patterns of seed dispersal between black‐casqued (a, c, e) and white‐thighed hornbill (b, d, f). Each seed dispersal simulation is based on a redistribution kernel that incorporates step lengths, turning angles and habitat selection of the respective hornbill species. Each simulation originated at the crown of a fruiting tree on the study site (represented by red points); combinations of hornbills and trees include *Staudtia kamerunensis* (a, b), *Xylopia hypolampra* (c, d) and *Maesopsis eminii* (e, f). Resulting patterns of seed dispersal are based on a gamma distribution of gut passage time along the length of 100 hornbill movement simulations. All seed deposition maps are plotted over a 1 m canopy height map modified to enhance visual clarity. GPT = Gut passage time, in minutes, ± standard error.

We generated a spatially explicit model of seed deposition probabilities for three species of hornbill‐dispersed trees occurring within the Bouamir Research Site, using canopy height, vertical complexity index, habitat type (swamp vs. non‐swamp) and the distribution of step lengths and turn angles as predictors (Figure 5). We omitted both ‘distance to canopy gap’ variables because they were not significant predictors of either species' movements at the population level. In evaluating the underlying movement models, we saw concordance between the values at used locations and those of simulated locations (Figure S7). The intensity (*λ*) of the Poisson point process underlying each model represents the average probability of seed dispersal per 10 m pixel and ranged from 7.33 · e^−5^ for *Staudtia kamerunensis* dispersed by white‐thighed hornbill (Figure 5b), to 1.31 · e^−4^ for *Maesopsis eminii* dispersed by either hornbill species (Figure 5e,f). The median simulated dispersal distances ranged from 457.59 to 687.85 m for hornbill‐tree species pairings (*n* = 6) and the maximum simulated dispersal distance ranged from 2391.70 to 2972.31 m (Figure S8).

## 4 DISCUSSION

Our results show that vegetation structure plays an important role in the movement decisions of seed‐dispersing hornbills and resulting spatial patterns of seed dispersal. Black‐casqued and white‐thighed hornbills prefer tall canopies but individuals of both species vary in their attraction to canopy gaps. These findings suggest that the 52 species of trees known to be dispersed by hornbills will likely arrive at areas with greater canopy height, and that variation in movement behaviour among individual hornbills may increase the possibility of seeds dispersing to areas suitable for germination. Although relatively small sample sizes of hornbills introduce some limitations to this study, the strength of selection for attributes of 3D vegetation structure indicate strong individual‐ and population‐level patterns. The results of this study point to the importance of 3D vegetation structure in shaping seed dispersal capabilities of tropical trees, an ecological process that shapes vegetation structure in turn.

Canopy height was an important driver of habitat selection by hornbills, which select fruits from trees that occupy the upper canopy of Cameroon's rainforests (Hardesty & Parker, 2002). The fruiting trees that attract hornbills tend to be among the tallest in the landscape, as slow‐growing trees characteristic of mature forests (Sonké & Couvreur, 2014; Whitney et al., 1998). Tall trees can also indicate potential nesting habitat because hornbills require large tree cavities, and nesting trees tend to be taller than surrounding trees (Stauffer & Smith, 2004). Canopy height is typically associated with vertical vegetation complexity (Gouveia et al., 2014). However, we did not find a strong correlation between these variables, and hornbills showed a broader variation of habitat selection in relation to vertical vegetation complexity. Because hornbills rarely descend below the upper canopy, where denser vegetation can restrict flight paths, it is possible that individual birds that selected for greater vertical vegetation complexity—including all five white‐thighed hornbills—might not be selecting for vegetation structure in the vertical column, but instead associating with structurally complex habitats, such as transitional habitats between forest and swamp or inselberg grasslands.

Three‐dimensional vegetation structure influences the behaviour of animals in a landscape by indicating reward and risk (Wittemyer et al., 2019). It is difficult to measure all landscape characteristics that may influence animal behaviour, so we encourage researchers to formulate hypotheses based on observations of the study species. In this study, we found that individuals of both hornbill species showed positive and negative selection for distance to canopy gaps of large (≥500 m^2^) and small areas (≥50 m^2^). Gap edge specialists may seek fruits of trees that are associated with inselberg edges, such as *Lannea welwitschii*, *Eribromum oblongum* and *Maesopsis eminii* (Whitney & Smith, 1998), or perhaps early colonizing tree species that produce fruits abundantly in canopy gaps, such as *Musanga cecropioides*. Canopy gaps could also present hornbills with an energy‐expensive crossing or expose them to one of their main predators, the crowned eagle (*Stephanoaetus coronatus*; Rainey et al., 2004). Gap‐avoiding hornbills may respond to the risks of visiting canopy gaps or the potential energy costs of moving across them (Davies et al., 2019; Gaynor et al., 2019). It might be expected that canopy gaps increase the energetic costs of flight, while taller canopies help decrease them. Still, we found no 3D structural attributes that predicted the overall dynamic body acceleration (ODBA) of hornbills, a proxy for energy use. Hornbills that did not move preferentially towards or away from canopy gaps may use an optimal foraging strategy that balances predation risk with the rewards of finding food (Abrahms et al., 2021). Taken together, these results suggest that hornbills can routinely deposit seeds in canopy gaps, provided perches are available.

Black‐casqued and white‐thighed hornbills are thought to be functionally similar as seed dispersers, given the extensive overlap in their diets (Whitney et al., 1998; Whitney & Smith, 1998). However, black‐casqued hornbills prefer *Raphia* palm‐dominated swamps over other habitat types during hotter times of day, while white‐thighed hornbills appear to avoid them altogether. This finding highlights an important niche difference between related species that can lead to a functional difference in seed dispersal beyond the differences in gut passage times of seeds. It is not immediately clear why white‐thighed hornbills avoid *Raphia* swamps, because both species have been observed gathering mud from swamps for their cavity nests during the onset of the breeding season (May–July) and consuming fruits of *Raphia monbuttorum* (Whitney et al., 1998). However, some white‐thighed hornbills may not have a large enough gape to consume the fruits (Whitney et al., 1998; Whitney & Smith, 1998). Black‐casqued hornbills are larger than white‐thighed and behaviorally dominant at fruiting trees (French & Smith, 2005). This behavioural hierarchy could extend to *Raphia* palm fruits or perhaps resting locations with cool temperatures and low predation risk. Indeed, we found that black‐casqued hornbills exhibited their lowest activity levels within swamp habitats. These findings shed light on the importance of *Raphia* swamps for black‐casqued hornbills, especially as climate change brings greater temperature and precipitation extremes to the region (Réjou‐Méchain et al., 2021). An interesting avenue for further research would be to explore how temperature—mediated by vegetation structure—influences habitat and nest site selection of hornbills.

Three‐dimensional vegetation structure is known to influence animal distributions, behaviour and niche differences, but few studies extend the importance of 3D vegetation structure to ecological functions performed by animals (Davies & Asner, 2014). In this paper, we demonstrated how 3D vegetation structure can modulate seed dispersal patterns by influencing animal dispersers' behaviour. Characterizing 3D structure with UAV‐LiDAR was easier and less costly than airborne LiDAR and enabled more granular descriptions of vertical and horizontal complexity that revealed differences in habitat selection between species and among individuals. There is growing interest in the predictive power of Step Selection Analyses, a standard method to analyse habitat selection of animals based on movement data (Potts et al., 2022). Recent advances in simulating animal movements based on habitat selection (Signer et al., 2024) enabled this paper's seed dispersal modelling framework. Mechanistic models of seed dispersal are becoming increasingly detailed in predictions of the spatial distribution of seed deposition probabilities (Borah & Beckman, 2022; Kleyheeg et al., 2017; Nield et al., 2020; Treep et al., 2021; Van Toor et al., 2019). To our knowledge, we present the first model that links coefficients from an iSSA with gut passage times to predict spatial patterns of seed deposition. This framework enables practitioners to include any variables of interest that influence habitat selection and movement behaviour of seed dispersers in predictions of seed dispersal patterns.

Seed dispersal gives rise to the intricate 3D structure of tropical rainforests, which creates heterogeneity in vertical and horizontal space for the most diverse biological communities on Earth. These ecosystems are threatened by myriad factors, including logging, mining, infrastructure expansion and commercial agriculture (Barlow et al., 2018). While hornbills show clear patterns of habitat selection in a mature rainforest, they encounter human‐dominated landscapes when they fly long distances during food‐lean times and some populations rely on forests and swamps near rural villages to survive and reproduce (Chasar et al., 2014). Across the tropics, human pressures curtail animal movements and reduce seed dispersal distances (Tucker et al., 2021). We have built a predictive model that can be used to understand how changes in 3D structure can affect seed dispersal, an important aspect of ecosystem structure and function. We encourage the use of this framework to characterize seed dispersal in other systems, as well as other ecosystem subsidies distributed by animals (Ellis‐Soto et al., 2021). Given the role of natural seed dispersal in forest restoration, the modelling framework presented in this paper may also serve as a useful tool for proactive ecosystem management (Estrada‐Villegas et al., 2023). Ecosystem functioning arises from the interactions among primary producers, consumers and abiotic factors (Schmitz, 2010; Schmitz et al., 2018). An intimate understanding of these interactions is necessary to predict the future of ecosystems in response to global climate and land use change.

## AUTHOR CONTRIBUTIONS

Nicholas J. Russo, António Ferraz, Nicolas Barbier, Martin Wikelski, Elsa M. Ordway, Sassan Saatchi and Thomas B. Smith conceived the ideas and designed methodology; Nicholas J. Russo, Docas L. Nshom and Nicolas Barbier collected the data; Nicholas J. Russo and Michael J. Noonan analysed the data, Nicholas J. Russo led the writing of the manuscript. All authors contributed critically to the drafts and gave final approval for publication.

## CONFLICT OF INTEREST STATEMENT

The authors have no conflicts of interest to declare.

## STATEMENT ON INCLUSION

The authors hail from several different countries, including the country where data were collected. We engaged local communities by hiring local guides for all data collection, presenting results and seeking feedback on research methods and implications.

## Supporting information


**Table S1.** Covariates included in model selection for integrated Step Selection Analysis.
**Table S2.** Pearson's correlation coefficient for each pairing of covariates included in habitat selection models.
**Table S3.** Predictors of black‐casqued hornbill activity—measured using Overall Dynamic Body Acceleration—ranked using AICc.
**Table S4.** Predictors of white‐thighed hornbill activity—measured using Overall Dynamic Body Acceleration—ranked using AICc.
**Figure S1.** Sampling period of all 21 hornbills tracked over the course of this study, where each point represents a single day of tracking.
**Figure S2.** Teflon harness removed from a recaptured white‐thighed hornbill.
**Figure S3.** Hornbill selection for habitat variables at 10 m resolution, based on 10 random steps, including (A) Canopy height, (B) Vertical complexity, (C) Distance to small canopy gaps (50 m^2^), (D) Distance to large canopy gaps (500 m^2^), (E) Swamp habitat and (F) The interaction between swamp selection and ambient temperature. Covariates included in the habitat selection‐free movement kernel of iSSAs include (G) Step length and (H) Turn angle.
**Figure S4.** Hornbill selection for habitat variables at 10 m resolution, based on 100 random steps, including (A) Canopy height, (B) Vertical complexity, (C) Distance to small canopy gaps (50 m^2^), (D) Distance to large canopy gaps (500 m^2^), (E) Swamp habitat and (F) The interaction between swamp selection and ambient temperature. Covariates included in the habitat selection‐free movement kernel of iSSAs include (G) Step length and (H) Turn angle.
**Figure S5.** Predicted ODBA with respect to temperature for (A) Black‐casqued and (B) White‐thighed hornbill based. Smoothed trendlines are based on a generalized linear mixed‐effects model that treats individual hornbill ID as a random effect, with 95% confidence intervals based on predictions conditioned on the fixed effects.
**Figure S6.** Predicted ODBA with respect to swamp habitat for black‐casqued hornbill.
**Figure S7.** Density plots comparing the distributions of covariates at simulated hornbill locations used in seed dispersal models based on (A, C, E) white‐thighed and (B, D, F) black‐casqued hornbill movements.
**Figure S8.** Histograms representing displacement distances based on simulated seed dispersal events.

## Data Availability

Data and code used to generate results are available from Figshare https://doi.org/10.6084/m9.figshare.25857385.v1 (Russo, 2024). Hornbill movement data are available on Movebank (movebank.org, study name “Hornbill e‐obs Cameroon”, study ID 2016993973).
