# Supplementary material for: Three‐dimensional vegetation structure drives patterns of seed dispersal by African hornbills
Source: J Anim Ecol. 2024 Oct 18;93(12):1935–46. doi: 10.1111/1365-2656.14202 (PMC11615260; doi:10.1111/1365-2656.14202)
Supplement: Supplementary file 1 — Table S1. Covariates included in model selection for integrated Step Selection Analysis. Table S2. Pearson's correlation coefficient for each pairing of covariates included in habitat selection models. Table S3. Predictors of black‐casqued hornbill activity—measured using Overall Dynamic Body Acceleration—ranked using AICc. Table S4. Predictors of white‐thighed hornbill activity—measured using Overall Dynamic Body Acceleration—ranked using AICc. Figure S1. Sampling period of all 21 hornbills tracked over the course of this study, where each point represents a single day of tracking. Figure S2. Teflon harness removed from a recaptured white‐thighed hornbill. Figure S3. Hornbill selection for habitat variables at 10 m resolution, based on 10 random steps, including (A) Canopy height, (B) Vertical complexity, (C) Distance to small canopy gaps (50 m2), (D) Distance to large canopy gaps (500 m2), (E) Swamp habitat and (F) The interaction between swamp selection and ambient temperature. Covariates included in the habitat selection‐free movement kernel of iSSAs include (G) Step length and (H) Turn angle. Figure S4. Hornbill selection for habitat variables at 10 m resolution, based on 100 random steps, including (A) Canopy height, (B) Vertical complexity, (C) Distance to small canopy gaps (50 m2), (D) Distance to large canopy gaps (500 m2), (E) Swamp habitat and (F) The interaction between swamp selection and ambient temperature. Covariates included in the habitat selection‐free movement kernel of iSSAs include (G) Step length and (H) Turn angle. Figure S5. Predicted ODBA with respect to temperature for (A) Black‐casqued and (B) White‐thighed hornbill based. Smoothed trendlines are based on a generalized linear mixed‐effects model that treats individual hornbill ID as a random effect, with 95% confidence intervals based on predictions conditioned on the fixed effects. Figure S6. Predicted ODBA with respect to swamp habitat for black‐casqued hornbill. Figure S7. Density plot [file JANE-93-1935-s001.docx]

**Supplemental Material**

**Table S1:** Covariates included in model selection for integrated Step Selection Analysis

| **Covariate** | **Definition** | **Units** | **Spatial resolution** |
| --- | --- | --- | --- |
| Canopy Height | Height of first LiDAR return (scaled and centered) | Meters | 10 m |
| Vertical Complexity Index | A fixed normalization of the entropy function, based on the 3D point cloud (scaled and centered) | Unitless | 10 m |
| Distance to gap 50 m^2^ | Distance to gap of minimum size 50 m^2^, 5 m height threshold (scaled and centered) | Meters | 10 m |
| Distance to gap 500 m^2^ | Distance to gap of minimum size 500 m^2^, 5 m height threshold (scaled and centered) | Meters | 10 m |
| Swamp | Habitat class defined as swamp, all other classes (*terra firme* forest, inselberg) taking on zero | Unitless (binary) | 10 m |
| Temperature:Swamp | Interaction between ambient temperature (scaled and centered) and habitat class defined as swamp | NA | 10 m |
| log(Step length + 1) | Distance between two successive GPS locations + 1 m—to account for step lengths of 0—and log-transformed | Meters | NA |
| cos(Turn angle) | Cosine of the angle between two successive GPS locations | Radians | NA |
| Step ID | Stratum consisting of a selected step (n=1) and randomly generated steps (n=10) | NA | NA |

**Table S2:** Pearson’s correlation coefficient for each pairing of covariates included in habitat selection models

|  | CanopyHeight10m | VCI | dist2gap50 | dist2gap500 | Swamp |
| --- | --- | --- | --- | --- | --- |
| CanopyHeight10m | 1 | 0.472887 | 0.223225 | 0.124654 | -0.24559 |
| VCI | 0.472887 | 1 | 0.179814 | 0.179794 | -0.21498 |
| dist2gap50 | 0.223225 | 0.179814 | 1 | 0.200184 | -0.04672 |
| dist2gap500 | 0.124654 | 0.179794 | 0.200184 | 1 | 0.000122 |
| Swamp | -0.24559 | -0.21498 | -0.04672 | 0.000122 | 1 |

**Table S3:** Predictors of black-casqued hornbill activity—measured using Overall Dynamic Body Acceleration—ranked using AICc. The models shown are limited to those that contribute to a cumulative weight of 99%. ***k*** = number of parameters; **log(ʆ)** = log-likelihood; **AIC_c_**= Akaike Information Criterion corrected for small sample size; **Δ*_i_*  =** difference in AIC_c_ units between model *i* and the top model; ***w*_i_** = weight of model *i*; $\sum_{\boldsymbol{i=1}}^{\boldsymbol{n}} \boldsymbol{w}_{\boldsymbol{i}}$ = cumulative weight up to and including model *i*.

| **Model** | ***k*** | **log(ʆ)** | **AIC_c_** | **Δ*_i_*** | ***w_i_*** | $\sum_{\boldsymbol{i=1}}^{\boldsymbol{n}} \boldsymbol{w}_{\boldsymbol{i}}$ |
| --- | --- | --- | --- | --- | --- | --- |
| Temp + Swamp | 5 | 1829.194 | -3648.39 | 0 | 0.968117 | 0.968117 |
| Temp+ Swamp + dist2gap50 | 6 | 1825.983 | -3639.96 | 8.423388 | 0.014349 | 0.982466 |
| Temp | 4 | 1823.742 | -3639.48 | 8.904432 | 0.011281 | 0.993747 |

**Table S4:** Predictors of white-thighed hornbill activity—measured using Overall Dynamic Body Acceleration—ranked using AICc. The models shown are limited to those that contribute to a cumulative weight of 99%. ***k*** = number of parameters; **log(ʆ)** = log-likelihood; **AIC_c_**= Akaike Information Criterion corrected for small sample size; **Δ*_i_*  =** difference in AIC_c_ units between model *i* and the top model; ***w*_i_** = weight of model *i*; $\sum_{\boldsymbol{i=1}}^{\boldsymbol{n}} \boldsymbol{w}_{\boldsymbol{i}}$ = cumulative weight up to and including model *i*.

| **Model** | ***k*** | **log(ʆ)** | **AIC_c_** | **Δ*_i_*** | ***w_i_*** | $\sum_{\boldsymbol{i=1}}^{\boldsymbol{n}} \boldsymbol{w}_{\boldsymbol{i}}$ |
| --- | --- | --- | --- | --- | --- | --- |
| Temp | 4 | -458.593 | 925.188 | 0 | 0.92302 | 0.92302 |
| Temp + VCI | 5 | -460.669 | 931.3419 | 6.153862 | 0.042552 | 0.965572 |
| Temp + dist2gap500 | 5 | -461.902 | 933.8091 | 8.621047 | 0.012393 | 0.977965 |
| Temp + Swamp | 5 | -462.003 | 934.0114 | 8.82332 | 0.011201 | 0.989166 |
| Temp+ Canopy Height | 5 | -463.134 | 936.2721 | 11.08402 | 0.003617 | 0.992783 |


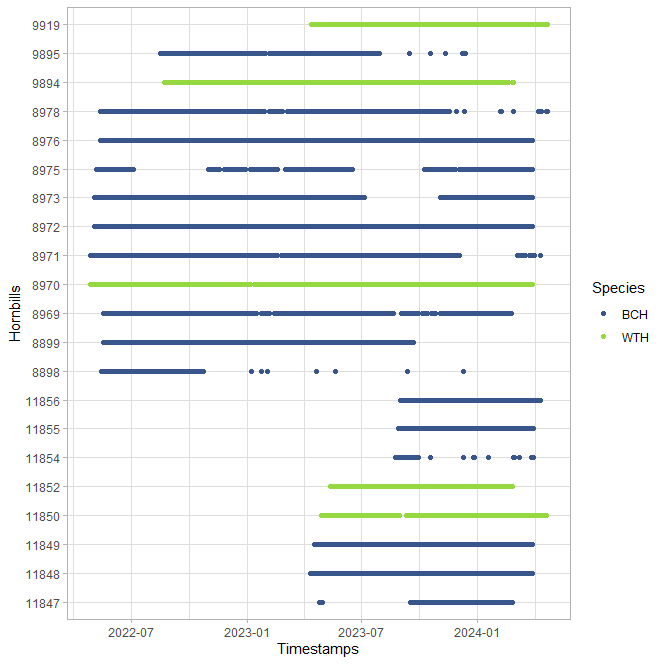


**Figure S1:** Sampling period of all 21 hornbills tracked over the course of this study, where each point represents a single day of tracking. BCH = Black-casqued hornbill; WTH = white-thighed hornbill.


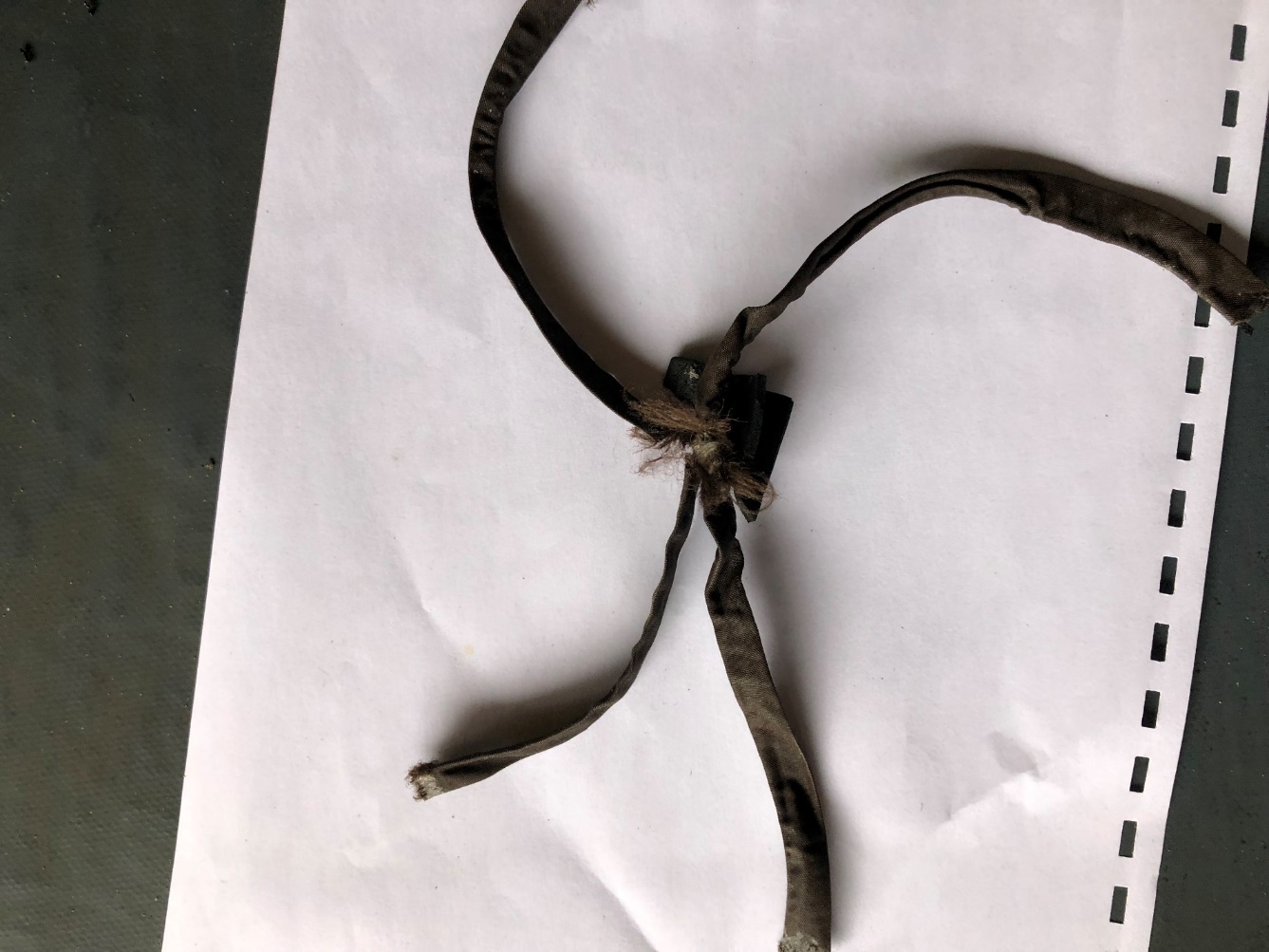


**Figure S2:** Teflon harness removed from a recaptured white-thighed hornbill.

Assessment: We recaptured a male white-thighed hornbill on August 17^th^, 2021, 15 days after its initial capture. To our knowledge, it is the only hornbill ever to be captured a second time in Cameroon. This event provided an opportunity to assess the impact of the Teflon harness on the bird’s body. After removing the harness, we noted rougher skin patches underneath the Teflon ribbon straps compared to the rest of bird’s body, but no injuries. We found evidence of the bird attempting to remove the harness at the center attachment point over the breast; both shoulder straps were worn nearly to detachment. The trammel line reinforcing the interior of the harness was undamaged and may have prevented the bird from removing the harness completely. After this assessment, we began to shape the Neoprene patch over the breast so that it aligned completely with the Teflon straps and provided less surface area for the bird to manipulate with its bill.


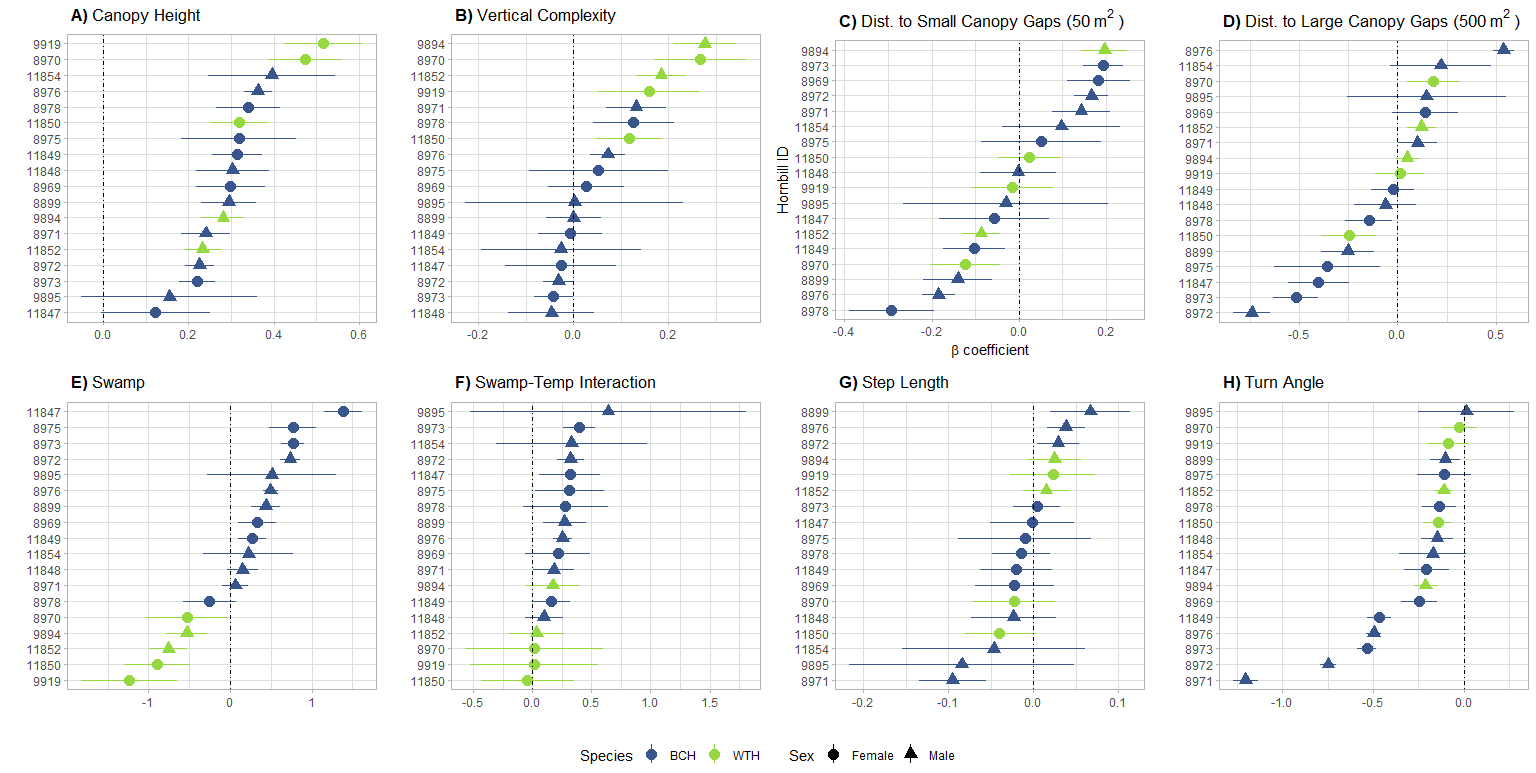


**Figure S3:** Hornbill selection for habitat variables at 10 m resolution, based on 10 random steps, including **A)** Canopy height, **B)** Vertical complexity, **C)** Distance to small canopy gaps (50 m^2^), **D)** Distance to large canopy gaps (500 m^2^), **E)** Swamp habitat, and **F)** The interaction between swamp selection and ambient temperature. Covariates included in the habitat selection-free movement kernel of iSSAs include **G)** Step length and **H)** Turn angle. Points represent coefficients and lines represent 95% confidence interval for each individual hornbill in the study. (BCH = Black-casqued hornbill, WTH = White-thighed hornbill). The dotted line at x=0 in each plot represents no selection. Note that the range of values in the x and y axes differs for each plot.


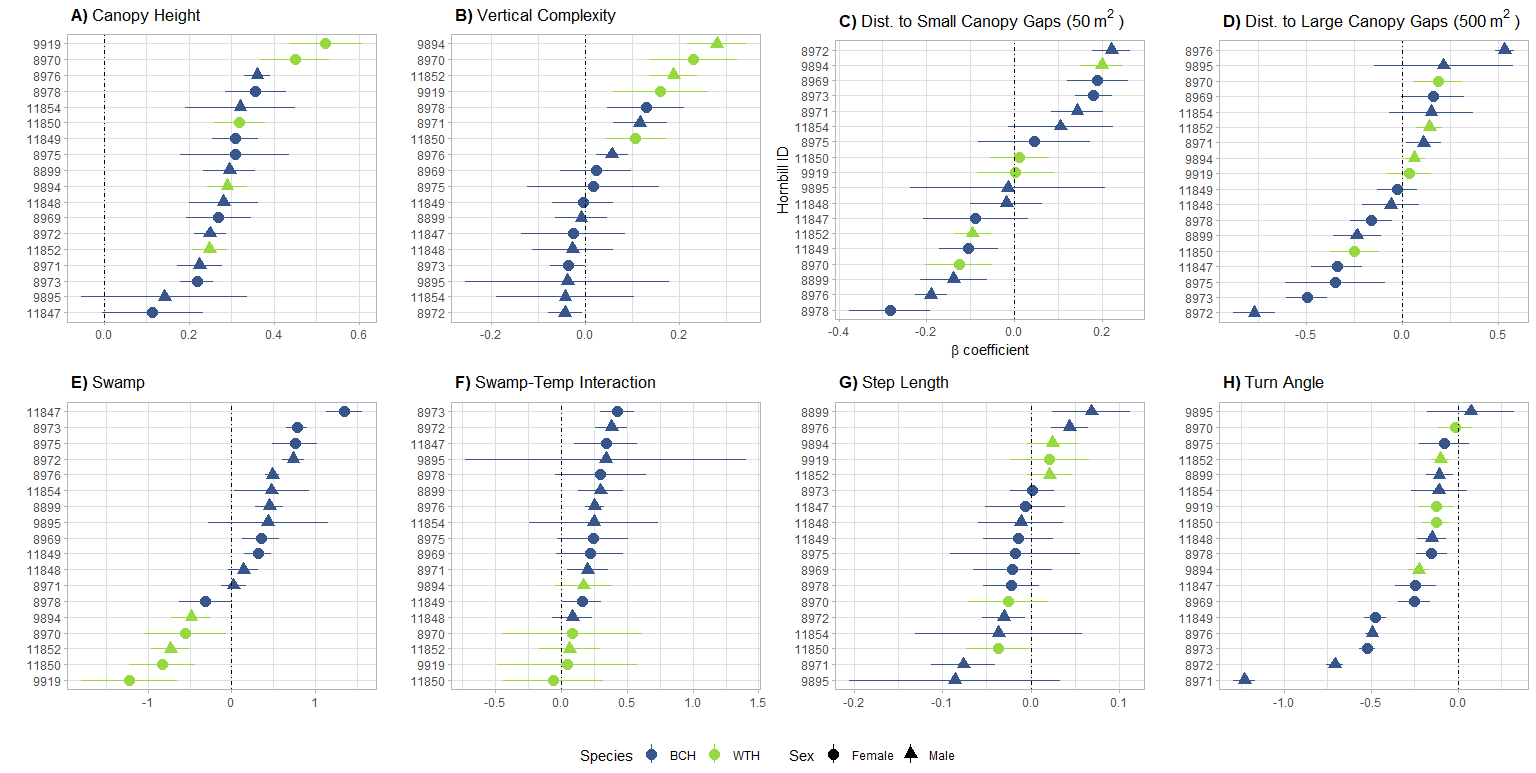


**Figure S4:** Hornbill selection for habitat variables at 10 m resolution, based on 100 random steps, including **A)** Canopy height, **B)** Vertical complexity, **C)** Distance to small canopy gaps (50 m^2^), **D)** Distance to large canopy gaps (500 m^2^), **E)** Swamp habitat, and **F)** The interaction between swamp selection and ambient temperature. Covariates included in the habitat selection-free movement kernel of iSSAs include **G)** Step length and **H)** Turn angle. Points represent coefficients and lines represent 95% confidence interval for each individual hornbill in the study. (BCH = Black-casqued hornbill, WTH = White-thighed hornbill). The dotted line at x=0 in each plot represents no selection. Note that the range of values in the x and y axes differs for each plot.


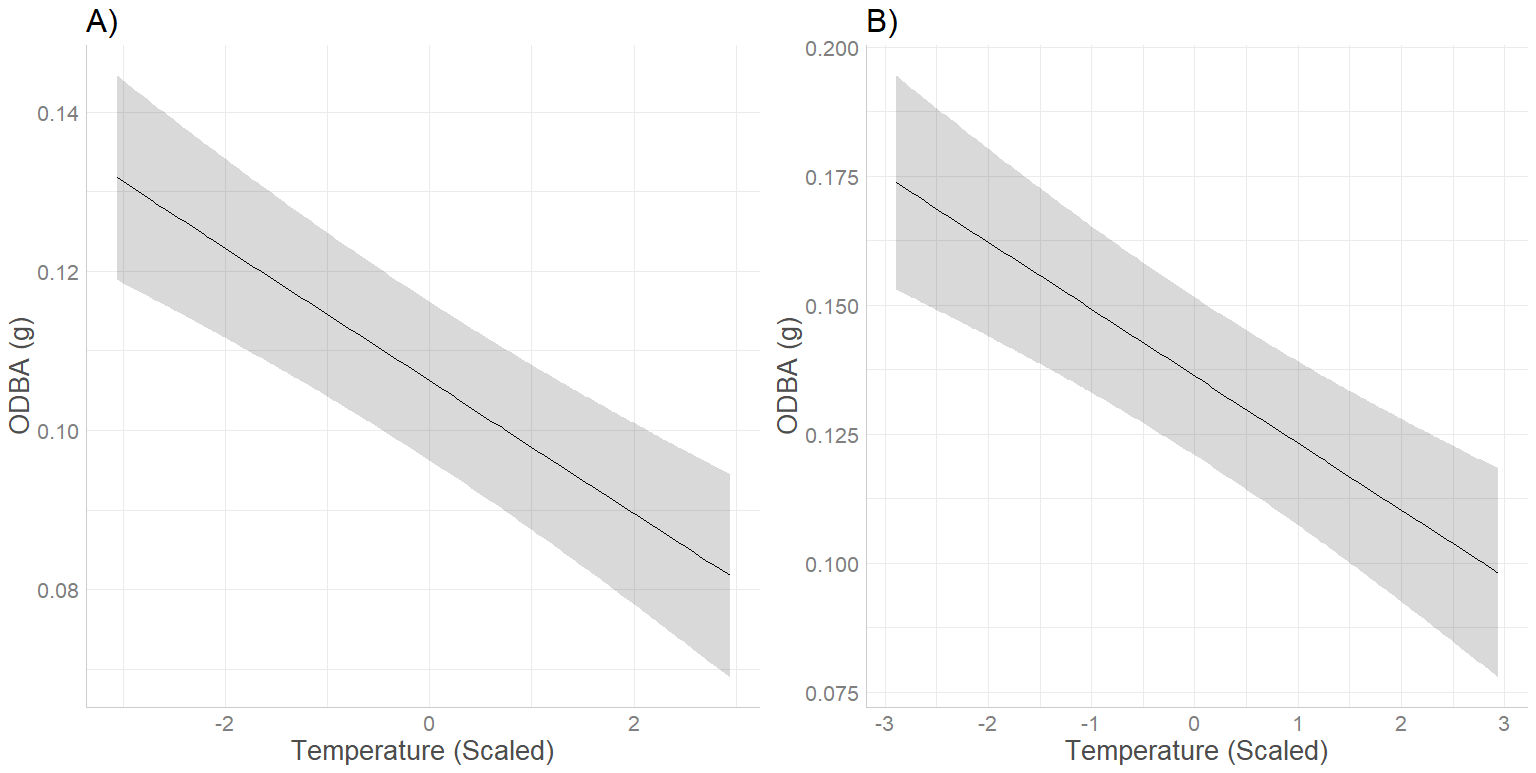


**Figure S5:** Predicted ODBA with respect to temperature for **A)** Black-casqued and **B)** White-thighed hornbill. Smoothed trendlines are based on a generalized linear mixed-effects model that treats individual hornbill ID as a random effect, with 95% confidence intervals based on predictions conditioned on the fixed effects.


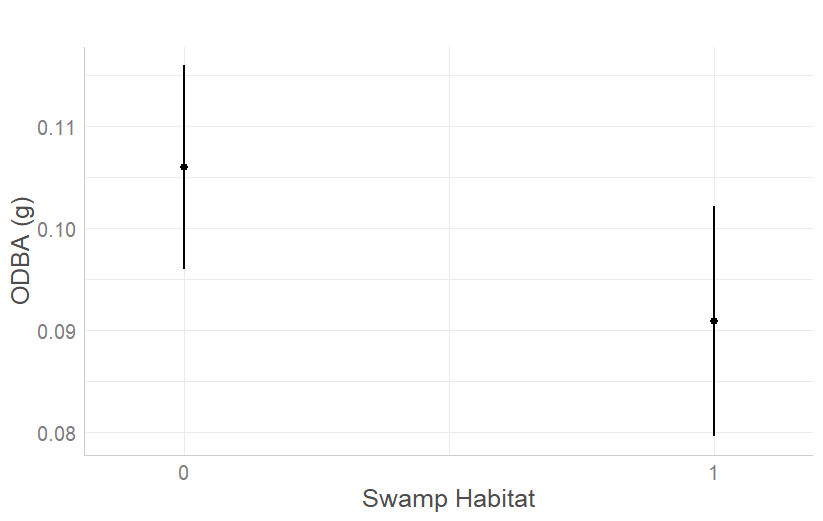


**Figure S6:** Predicted ODBA with respect to swamp habitat for black-casqued hornbill. Estimates and 95% confidence intervals are based on a generalized linear mixed effects model that treats

individual hornbill ID as a random effect.


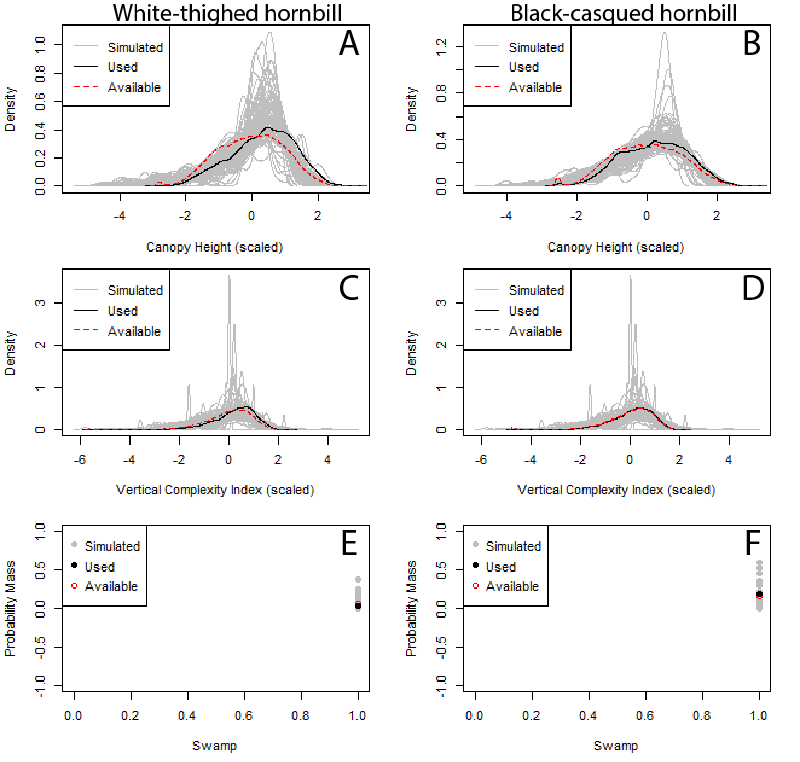


**Figure S7:** Density plots comparing the distributions of covariates at simulated hornbill locations used in seed dispersal models based on (**A, C, E**) white-thighed and (**B, D, F**) black-casqued hornbill movements. “Used” and “available” locations represent the ends of observed and randomly generated movement steps, respectively. As in used-habitat calibration plots, note that the lines for “used” habitat fall within the distribution of lines for 100 simulated predictions.


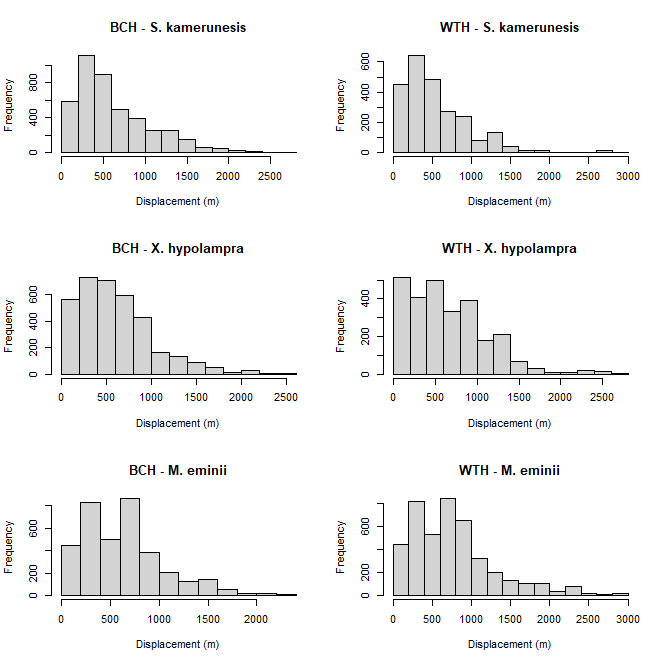


**Figure S8:** Histograms representing displacement distances based on simulated seed dispersal events. Hornbill-tree pairings match those shown in Figure 5 (BCH = Black-casqued hornbill; WTH = White-thighed hornbill).
